# Seasonal Variation of Rhegmatogenous Retinal Detachment in Greece: Temperature, Humidity, and Atmospheric Pressure as Risk Factors

**DOI:** 10.7759/cureus.84454

**Published:** 2025-05-20

**Authors:** Christos Tooulias, Dimitrios Papaconstantinou, Konstantinos Droutsas, Angeliki Androu, Paraskevi Theofilou, Panagiotis Theodossiadis, Panagiotis Stavrakas, Ilias Georgalas

**Affiliations:** 1 Second Department of Ophthalmology, Attikon University General Hospital, National and Kapodistrian University of Athens, Athens, GRC; 2 First Department of Ophthalmology, G. Gennimatas Hospital, National and Kapodistrian University of Athens, Athens, GRC; 3 Department of Ophthalmology, University of Patras, Patras, GRC

**Keywords:** air temperature, atmospheric pressure, greece, humidity, rhegmatogenous retinal detachment (rrd)

## Abstract

Background

This study aims to investigate the seasonal incidence of rhegmatogenous retinal detachment (RRD) in Greece, evaluating factors such as average temperature, relative humidity, and atmospheric pressure as potential risk factors for the observed patterns.

Methodology

A total of 363 patients diagnosed with RRD during a four-year period (January 1, 2020, to December 31, 2023) were analyzed retrospectively. Climate data for the present study were obtained from the records of the National Meteorological Service of Greece. The meteorological parameters included in the study were average temperature (°C), humidity (%), average atmospheric pressure (hPa), and monthly duration of sunshine (hours). The correlation of the above meteorological parameters with the occurrence of retinal detachment was analyzed both univariately (independent correlation of each meteorological parameter with retinal detachment) and multivariately (combined correlation of the meteorological parameters with retinal detachment).

Results

A statistically significant relationship was observed between the incidence of RRD and average temperature, average relative humidity, and average duration of sunshine, as indicated by the chi-square test (p < 0.005). However, no correlation was observed between the incidence of RRD and average atmospheric pressure.

Conclusions

This is the first epidemiological study conducted in Greece to assess the influence of meteorological and seasonal factors on the incidence of RRD. Our results indicate that the incidence of RRD in the central region of Greece is associated with a significant seasonal pattern, which can be attributed to average temperatures, humidity levels, and hours of sunlight.

## Introduction

Rhegmatogenous retinal detachment (RRD) constitutes a serious ophthalmologic condition characterized by the separation of the neurosensory retina from the underlying retinal pigment epithelium (RPE) due to the presence of a retinal tear. This tear allows liquefied vitreous humor to enter the subretinal space, accumulating between the retina and the RPE, leading to retinal detachment. If left untreated, RRD can result in progressive vision loss and potentially permanent blindness. The development of RRD is often influenced by both anatomical changes in the vitreous humor and various risk factors, including age, ocular trauma, and pre-existing conditions such as high myopia [1].

The mechanism of RRD is closely related to the aging process of the vitreous humor. Over time, the vitreous undergoes liquefaction, a process known as vitreous syneresis, which leads to the formation of pockets of liquified vitreous. This process, combined with posterior vitreous detachment (PVD), increases the risk of retinal tears or breaks [2]. In PVD, the vitreous pulls away from its attachment points on the retina and, provided that strong adhesion sites exist, traction can create retinal tears. Once a tear forms, liquefied vitreous can pass through and accumulate under the retina, leading to detachment [3].

Several well-established risk factors for RRD include high myopia, previous ocular surgery such as cataract extraction, and ocular trauma. High myopia increases the risk of RRD due to the elongation of the eye, which can cause thinning of the retina, making it more prone to tearing [4]. Cataract surgery, particularly if complicated, can lead to PVD, which, in turn, may result in retinal breaks [5]. Additionally, patients with a family history of retinal detachment or with certain hereditary conditions, such as Stickler syndrome, are also at increased risk [6]. Other contributing factors include lattice degeneration [7] and certain systemic diseases, such as diabetes, which may affect the retinal microenvironment [8].

Seasonal variation in the incidence of RRD has been reported in various geographical regions, suggesting a possible influence of environmental factors on vitreous changes and retinal susceptibility [9]. Specifically, fluctuations in temperature, atmospheric pressure, and sunlight exposure may influence the behavior of the vitreous humor. Additionally, seasonal changes in the composition of the vitreous, which could affect its liquefaction and traction forces on the retina, have been hypothesized to contribute to variations in the incidence of RRD [10]. However, these associations have been understudied in Mediterranean countries such as Greece, where seasonal climate variations are pronounced. This study aims to investigate the seasonal incidence of RRD in Greece, focusing on potential changes in the biochemical composition of the vitreous humor and evaluating factors such as average temperature, relative humidity, and atmospheric pressure as potential risk factors for the observed patterns.

## Materials and methods

For this investigation, data for patients presenting with RRD to two tertiary hospitals during a four-year period (January 1, 2020, to December 31, 2023) were analyzed retrospectively. The research protocol was approved by the Scientific Council of the General University Hospital of Athens, G. Gennimatas (protocol number: 1920028074). The Declaration of Helsinki ethical principles for research involving humans were applied throughout the study. As a retrospective study, the ethics committee waived the need for written informed consent. This is a four-year prospective cohort study involving 363 patients with retinal detachment who visited the emergency department of the Second Ophthalmology Clinic of the Attikon University Hospital and the First University Ophthalmology Clinic of G. Gennimatas University Hospital, two of the largest university hospitals in Greece, whose ophthalmology departments receive and address the vast majority of vitreoretinal cases in Greece.

Patient eligibility criteria

Patients with fundoscopically proven retinal detachment who provided reliable information regarding the onset of relevant symptoms were included. In complex cases, such as those involving severe refractive media opacification, β-ultrasound could be used as an alternative to fundoscopy. All data were received after a chart and surgical records review by a designated author (CT) due to the retrospective design of this study. Cases where the perception of symptoms did not align with fundoscopic signs of chronicity (e.g., significant proliferative vitreoretinopathy (PVR)) were excluded from the study. We also excluded those who had undergone previous ocular surgery scientifically linked to RRD. This included cataract extraction surgery, vitrectomy, intravitreal anti-vascular endothelial growth factor injections, cortisone injections, or YAG laser capsulotomy in the study eye within six months before diagnosis. Finally, all cases with missing information either on disease characteristics (e.g., macula-on, macula-off, PVR) or on patients’ perspective regarding the onset of relevant symptoms, such as photopsia, floaters, or blurred vision, were also excluded.

Meteorological data

Climate data for the present study were obtained from the records of the National Meteorological Service of Greece. The seasons were defined as follows: winter (December, January, February), spring (March, April, May), summer (June, July, August), and autumn (September, October, November). Monthly meteorological parameter values used in this study were sourced from the network of weather stations operated by the National Observatory of Athens (EAA), specifically from the Athens Central Sector Station, located at an altitude of 50 m with a latitude of 23.71°E and a longitude of 37.97°N. The meteorological parameters included in the study were average temperature (°C), humidity (%), average atmospheric pressure (hPa), and monthly duration of sunshine (hours). The correlation of the above meteorological parameters with the occurrence of retinal detachment was analyzed both univariately (independent correlation of each meteorological parameter with retinal detachment) and multivariately (combined correlation of the meteorological parameters with retinal detachment).

Statistical analysis

We analyzed the variations in RRD frequency on a monthly and seasonal basis (eyes per month/eyes per season). A one-way analysis of variance (ANOVA) using SPSS Version 23.0 (IBM Corp., Armonk, NY, USA) was conducted to compare RRD frequency across months and seasons. The chi-squared test was employed to assess the relationship between RRD cases and seasonal or meteorological variables. A p-value of less than 0.05 was considered statistically significant.

## Results

Study sample characteristics

The total sample population included 363 patients diagnosed with RRD from January 2020 to December 2023. Men (n = 222; 61.3%) outnumbered women (n = 140; 38.7%) in our sample. The mean age was 69.05 ± 6.05 years (range = 50-82 years). The average male age was 69.50 ± 6.40 years (median = 69.5 years), ranging from 57 to 82 years, while the average female age was 68.34 ± 6.69 (median = 68 years), ranging from 50 to 81 years. There was no statistically significant difference in age distribution between the two sexes (p = 0.512). In addition, 178 (49.1%) eyes were left, while 184 (50.9%) were right eyes.

Monthly and seasonal variations in the surgical repair of RRD

**Figure 1:**
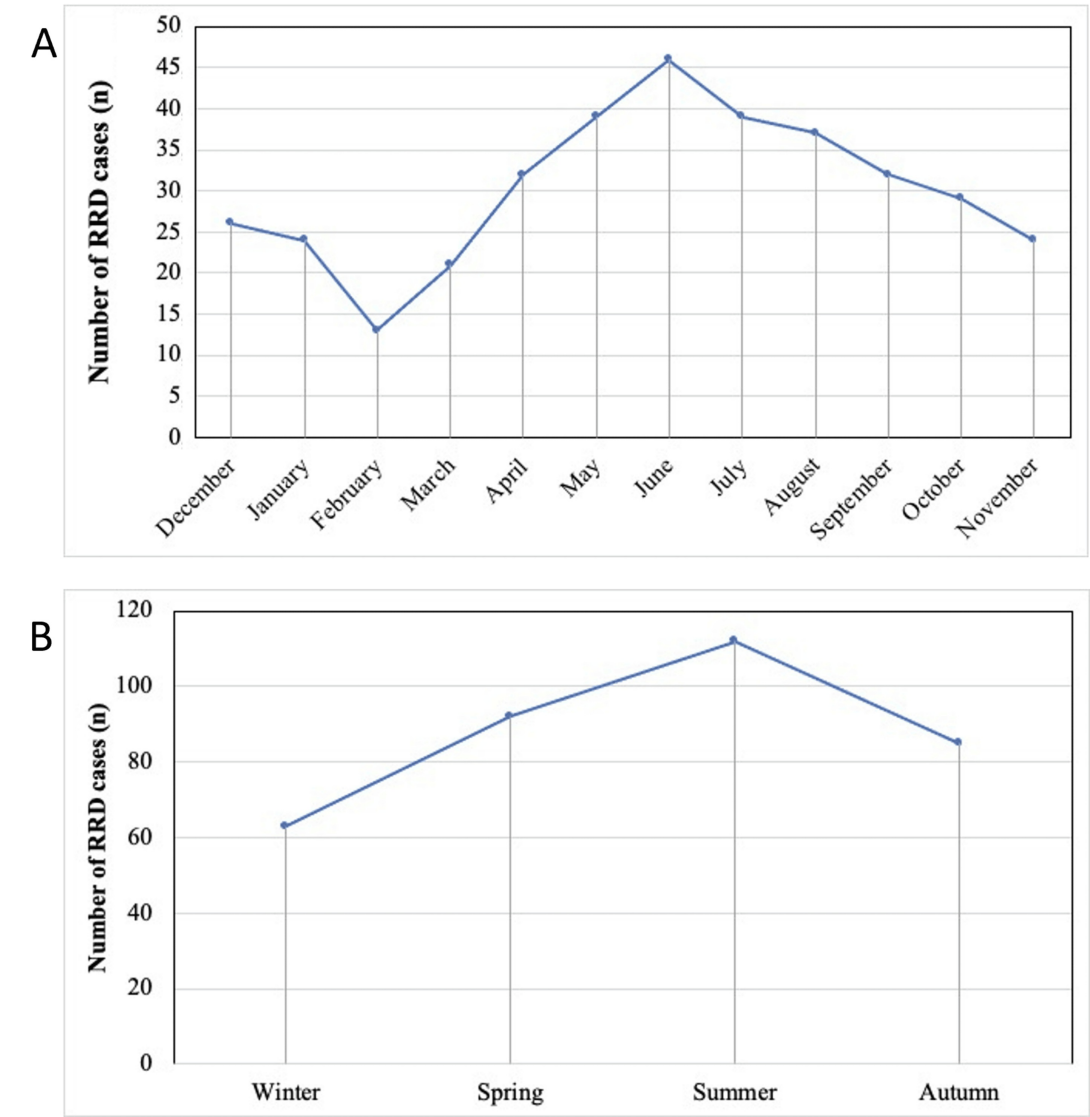
Monthly and seasonal variation in RRD incidence. (A) Monthly variation in RRD. (B) Seasonal variation in RRD. RRD: rhegmatogenous retinal detachment

The monthly incidence of retinal detachment (eyes per month) was the highest in June (n = 46) and the lowest in February (n = 13) (p < 0.05; comparing incidence across months) (Table 1, Figure 1).

**Table 1:** Number of RRD surgery cases per month from January 2020 to December 2023. RRD: rhegmatogenous retinal detachment

Year/Month	2020	2021	2022	2023	Number of cases	Percentage (%)
December	5	6	8	7	6.50	7.18
January	5	7	7	5	6.00	6.63
February	3	3	4	3	3.25	3.59
March	5	6	5	5	5.25	5.80
April	8	9	8	7	8.00	8.84
May	10	9	11	9	9.75	10.77
June	11	12	11	12	11.50	12.71
July	9	10	9	11	9.75	10.77
August	9	8	9	11	9.25	10.22
September	8	7	8	9	8.00	8.84
October	8	7	7	7	7.25	8.01
November	5	6	6	7	6.00	6.63

All three years analyzed exhibited a similar seasonal pattern across months (Table 1, Figure 1). Αll three years analyzed exhibited a similar seasonal pattern with no statistically significant difference in seasonal prevalence among the years (p = 0.778).

The seasonal incidence of retinal detachment (eyes per season) was the highest in summer (n = 112) and the lowest in winter (n = 63), with a statistically significant difference among seasons (p < 0.05) (Figure 1). The proportion of patients with “macula on” retinal detachment was 38.5%, while the percentage of patients with “macula off” retinal detachment was 61.5%. However, the mean seasonal frequency of these two groups did not differ significantly (p = 0.213). Finally, 51.7% of retinal detachment cases were associated with PVD, and the seasonal variation in the mean frequency of these cases was also not statistically significant (p = 0.271).

RRD prevalence according to meteorological variables

**Figure 2:**
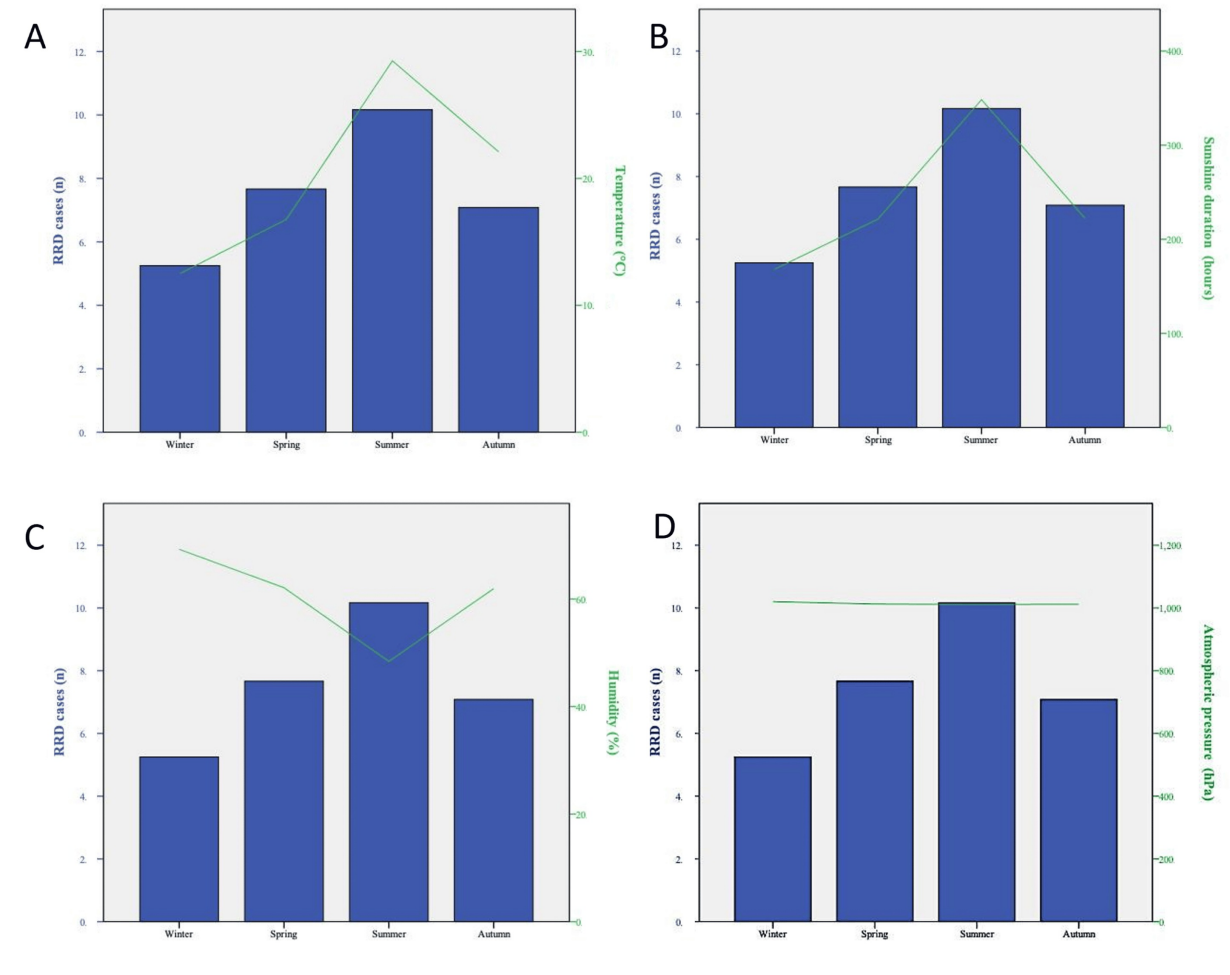
Analysis of RRD risk according to meteorological parameters: average seasonal RRD cases and average seasonal (A) temperatures (℃), (B) sunlight hours (hours), (C) humidity (%), and (D) average atmospheric pressure (hPa). RRD: rhegmatogenous retinal detachment

All meteorological parameters exhibited strong seasonality. Mean temperature and mean monthly sunshine duration were significantly higher during the summer months compared to the winter months (p < 0.001 and p = 0.001, respectively). The prevalence of RRD was the highest in summer when the average monthly temperature was between 25.7°C and 31.8°C and the hours of sunlight were between 294 and 363 hours per month. At the same time, the lowest prevalence of RRD was noted in winter when the average monthly temperature was between 10.4°C and 14.4°C (Figures 2A, 2B). Accordingly, humidity was significantly higher in the winter months compared to the summer months (p = 0.003). The prevalence of RRD was the highest when the humidity was 70.4%, while it was at its lowest when humidity levels were below 51.8% (Figure 2C). Finally, the distributions of average atmospheric pressure and RRD were not statistically significant (Figure 2D).

## Discussion

In this study, data from two public institutions were used to investigate the seasonality and potential influence of meteorological factors on the incidence of retinal detachment in the Greek population. The specific hospital institutions are geographically located in central Attica, a region characterized by a subtropical Mediterranean climate, according to the Köppen climate classification [11]. The main features of this climate are prolonged hot, dry summers and mild, wet winters.

A statistically significant relationship between the incidence of RRD and average temperature, average relative humidity, and average duration of sunshine was observed. However, no correlation was observed between the incidence of RRD and average atmospheric pressure. Specifically, the seasonal frequency of retinal detachment (eyes per season) was the highest in the summer (n = 112) and the lowest in the winter (n = 63) (p < 0.001). This result is in accordance with previous studies [12-19].

Weekers was the first to describe seasonality in the incidence of RRD in a series of 208 patients operated on in Liege, France, between 1930 and 1945 [20]. The author observed that 61% of RRD cases occurred during spring and summer and speculated that variations in meteorological factors were the most likely explanation for this phenomenon. In the years that followed, reports of seasonality increased, particularly in European and Asian countries, with several theories proposed to explain its occurrence.

In a retrospective study conducted in Pavia, Italy, with a 10-year follow-up period (n = 363), Ghisolfi et al. found a statistically significant association between quarterly radiation levels and the incidence of RRD [18]. They hypothesized that light radiation leads to metabolic overload, resulting in functional RPE deficiency. This potential phototoxic damage to the retina, RPE, and vitreous humor, caused by prolonged exposure to ultraviolet light during the summer, has been supported by several other studies [13,21]. Additionally, animal studies have shown that low temperatures help the vitreous remain more adherent to the retina, preventing posterior detachment [22]. Conversely, high temperatures have been associated with PVD in both animals [23] and humans [12]. More specifically, Rahman et al. evaluated the effects of ambient temperature, relative humidity, and radiation on the incidence of PVD over a two-year period. The authors reported a highly significant association between mean weekly temperature and the incidence of PVD, but no association was established for relative humidity or solar radiation [23].

Warm months are also associated with increased outdoor activity. As a result, heat-induced dehydration, exacerbated by physical activity and increased vitreous mobility, could contribute to the occurrence of PVD [24]. The restriction of outdoor activity during the COVID-19 lockdown provides additional supporting data. A study in France found a 41.6% decrease in the number of RRD surgeries performed during this period compared to the same period in 2019. In the four months following the lockdown, this number returned to normal [25], further suggesting that RRD was less frequent when the population was less physically active. Similar trends were observed in other countries, where studies also reported a decrease in RRD procedures during the global lockdown [26-27].

The role of temperature as a possible mechanism for the seasonality associated with retinal detachment was also confirmed by Lin et al. in a multicenter study conducted in Taiwan between 1999 and 2009, involving 23.818 eyes with RRD [15]. The authors observed that the monthly incidence of RRD in Taiwan follows a significant seasonal pattern, positively correlated with temperature and negatively correlated with atmospheric pressure. Furthermore, in the study by Barioulet et al., which examined the influence of meteorological factors on RRD incidence across metropolitan France, a statistically significant relationship was found between RRD incidence and average temperature in most urban areas, though no correlation was observed with other meteorological parameters, such as atmospheric pressure, sunshine duration, and relative humidity [27]. Laatikainen et al. evaluated 301 patients over a four-year period and reported more RRDs in the summer months compared to winter [28]. In contrast, a seven-year observational study from Kuwait found a higher incidence of RRD in winter months than in summer [29].

Our study also noted a statistically significant association between the occurrence of RRD and relative humidity. Specifically, the incidence of RRD was higher during the months with lower relative humidity (%). The role of relative humidity in the incidence of RRD was also assessed by Ghisolfi et al. in Italy (n = 363) [18], Mansour et al. in Lebanon (n = 211) [14], Lin et al. in Taiwan (n = 23,818) [15], and Prabhu et al. in India (n = 76) [16]. However, none of these studies were able to demonstrate a significant association, although Mansour et al. suggested that low humidity levels could be linked to RRD due to increased ocular itching. The frequency of eye rubbing due to allergies also rises in spring/summer, which may further contribute to a higher risk of RRD.

In addition to seasonality, analyzing intertemporal fluctuations is crucial for understanding the temporal evolution of retinal detachment incidence. In this aspect, our results did not show a significant increase in RRD incidence over the entire study period, as the annual differences were not statistically significant. However, previous studies in Europe, the United States, and Asia reported a significant increase in RRD cases, particularly among men, with the number of cases rising each year between January 2011 and December 2018 [30-32]. In a study by Shah et al. conducted in England, which evaluated the epidemiology of RRD, an increasing trend in hospital admissions for RRD was noted. The authors partly attributed this trend to the rising prevalence of diabetes mellitus, although they suggested that other factors were likely involved [33].

However, our analysis may be affected by biases, such as the potential for patients to have pursued medical assistance at other hospitals. The study also faces limitations due to the sample size and the potential biases inherent in retrospective designs. There was an absence of control for individual-level confounders such as smoking, comorbidities such as diabetes, and other similar factors. Age, sex, refractive error, or previous surgery as predisposing factors, some of which could also exhibit some seasonality, were not taken into account in this study for simplicity reasons. However, this constitutes a study limitation that needs to be addressed by future studies. Additionally, non-meteorological factors, such as annual events impacting lifestyle or physical activity, especially during the COVID-19 period, could affect seasonal variations in RRD incidence. Our study covers a four-year period, which provides a large time-frame for evaluating seasonal trends and inter-annual consistency. However, it is important to point out that at least part of the study period refers to the COVID-19 outbreak, when some changes in hospital policy or referral patterns could have influenced presentation numbers independently of the weather. To achieve more definitive results, increasing the sample size by including all cases is necessary. Therefore, an advanced database is required for a comprehensive evaluation of all cases. Finally, this study links aggregated weather data to patient presentation rates, rather than focusing on direct measurements of individual exposure to temperature or humidity. More laboratory-based studies are required to address this limitation.

This study provided a nationwide analysis with a sizable patient population, allowing for the evaluation of RRD seasonality. Despite its limitations, it is the first to assess the seasonality of RRD prevalence in Greece.

## Conclusions

This is the first epidemiological study conducted in Greece to assess the influence of meteorological and seasonal factors on the incidence of RRD. This study provided a nationwide analysis with a sizable patient population, allowing for the evaluation of RRD seasonality. Despite its limitations, and to our knowledge, it is the first to assess the seasonality of RRD prevalence in Greece. Our results indicate that the incidence of RRD in the central region of Greece is associated with a significant seasonal pattern, which can be attributed to average temperatures, humidity levels, and hours of sunlight. The highest incidence was noted in the summer, while the lowest was observed in the winter. Population-based data investigating the seasonal variation of RRD worldwide is scarce in the published literature. To date, the role of seasonal variation in RRD has been reported in several studies; however, few studies have reached statistically significant conclusions. Climatic parameters may be important for the development of RRD, and their identification is not only crucial for understanding its pathophysiology but also for risk awareness and the establishment of prevention strategies. Registries may provide some of this data, as they are particularly useful in the research of rare diseases such as RRD. Preventing and diagnosing RRD early on can be facilitated by an understanding of the effects of seasonal and meteorological factors. Further research into the pathophysiology of RRD is required to ascertain its precise etiology.
